# The Challenge of Heart Failure Discharge from the Emergency Department

**DOI:** 10.1007/s11897-012-0100-1

**Published:** 2012-07-15

**Authors:** Edwin C. Ho, Michael J. Schull, Douglas S. Lee

**Affiliations:** 1 Institute for Clinical Evaluative Sciences, Division of Cardiology, University Health Network, Room G-106, 2075 Bayview Avenue, Toronto, ON M4N 3M5 Canada; 2 Sunnybrook and Institute for Clinical Evaluative Sciences, and the Division of Emergency Medicine, Department of Medicine, University of Toronto, Toronto, Canada

**Keywords:** Heart failure, Emergency department, Risk stratification, Hospitalization, Hospital discharge

## Abstract

Acute decompensated heart failure is a common reason for presentation to the emergency department and is associated with high rates of admission to hospital. Distinguishing between higher-risk patients needing hospitalization and lower-risk patients suitable for discharge home is important to optimize both cost-effectiveness and clinical outcomes. However, this can be challenging and few validated risk stratification tools currently exist to help clinicians. Some prognostic variables predict risks broadly in those who are admitted or discharged from the emergency department. Risk stratification methods such as the Emergency Heart Failure Mortality Risk Grade and Acute Heart Failure Index clinical decision support tools, which utilize many of these predictors, have been found to be accurate in identifying low-risk patients. The use of observation units may also be a cost-effective adjunctive strategy that can assist in determining disposition from the emergency department.

## Introduction: Significance of Acute Heart Failure

The initial clinical encounter for acute decompensated heart failure (ADHF) often occurs in the emergency department (ED), followed frequently by hospitalization. Heart failure (HF) has become one of the leading reasons for hospitalization with over 1.1 million hospital admissions for the condition in the United States in 2006. The direct and indirect costs of HF are estimated to be approximately $40 billion in the United States alone [1].

The clinical course of HF patients who visit the ED is characterized by repeat visits to the ED, hospital readmissions, and high mortality [2–5]. United States Medicare data reported that patients who were hospitalized for HF in 1999–2000 had a 50 % all-cause readmission rate, 20 % HF readmission rate, and 31.4 % had died within 1 year [6]. Over 50 % of HF patients in this study revisited the ED within 3 months of discharge [6].

An important decision that must be made in the ED is whether to admit patients presenting with acute HF to an inpatient unit or to discharge them home. At the present time, these decisions are based primarily on clinical judgement, and are not necessarily guided by prognostic guidance. The variability in the results of clinical judgement is reflected by the differences in the crude discharge rates of HF patients from the ED. A multicenter study conducted in 20 hospitals in Spain showed an ED discharge rate of 32.7 % [7•]. Similarly, among patients diagnosed with acute HF in an ED in Alberta, Canada, approximately one third of these patients were not admitted to hospital [8]. Data from the Peer Review Organization Voluntary hospital association Initiative to Decrease Events (PROVIDE) for HF study described higher admission rates in the participating United States health care centers compared to the rates described in Canada and Spain [9].

There are many potential reasons why the discharge rates of ADHF patients to home from the ED may vary. These include variations in clinical judgement, perceived standard of care, local hospital or physician practice culture, potential medical-legal ramifications, and regional patterns of hospitalization [10]. Because some of these factors are more modifiable than others, tools to assist with clinical decision-making can potentially improve patient care. For example, Smith et al. [11] found that based on clinical judgement alone, physician estimation of the need for critical care management in ADHF was inaccurate. In this case, there was overestimation of the need for this level of care.

Prior studies have demonstrated the pitfalls of decision-making based solely on clinical grounds in the ED. An analysis of a population-based database of HF patients in Ontario, Canada reported that there was substantial overlap in the predicted mortality risks of HF patients who were discharged from the ED or admitted to hospital [12••]. Specifically, some patients who were discharged were at higher risk of 7-day death, while conversely, many hospitalized patients were low risk. When those with similar predicted risks of death were compared, those who were discharged had a higher observed risk of 90-day mortality. In the absence of clinical risk stratification to guide admission-discharge decisions, others have demonstrated that indiscriminate hospitalization of HF patients does not lead to a reduction in mortality or repeat ED visits [7•].

Ideally, patients who are low risk could be considered for discharge home without immediate hospitalization if they improve symptomatically. However, many low-risk patients are admitted to hospital because decision support methods that provide prognostic guidance have not been available. Hospitalizations and hospital readmissions are major contributors to the costs of HF care. Being better able to decide upon who needs hospital admission or can be discharged home may lead to improved utilization of health care resources and decreased costs. However, doing so will require that those who are discharged from the ED are provided rapid and appropriate follow-up care.

Studies of those who were discharged from the ED suggest that patients may return for acute medical care if a systematic approach to post-discharge follow-up care is not enabled. In one report, 61 % of patients discharged home from the ED with a diagnosis of ADHF experienced failure of outpatient therapy within 90 days. The median time to failure of therapy (defined as recurrent presentation with ADHF, hospitalization for ADHF or death) was 30 days [13]. Another study from Alberta, Canada found a significantly increased rate of repeat visits to the ED at 30 days and 1 year among those HF patients who were discharged home from the ED compared to those who were admitted [8]. In addition, the authors found that the all-cause mortality rate was also significantly lower in the admitted group.

While the optimal model of acute HF follow-up care has not been determined, early physician collaboration is an important component of the care regimen [14••]. In a propensity-matched study of patients who were discharged from the ED, those who received early collaborative care by a cardiac specialist and a primary care provider had substantially reduced mortality compared to those who were assessed by either type of physician alone [14••]. The risk of the composite of death, repeat ED visits, or hospitalization for any cause, was similarly reduced among those with collaborative care [14••]. Notably, the first physician visit occurred a median of 3 days post-discharge and 75 % of patients were seen within 7 days among those receiving collaborative care. In contrast, the first physician visit occurred a median of 5 days (75 % within 12 days) in the primary care only and a median of 9 days (75 % within 18 days) after the index ED discharge in the cardiology only groups. Uniformly, the worst outcomes were observed among those who were not assessed by either type of physician within 30 days.

## Prognostic Factors of HF Patients in the ED Setting

Although the ED is frequently the point of initial hospital contact of patients with acute HF, the ED has not been widely considered as an inception point from the standpoint of clinical registries. As a consequence, there is a paucity of evidence that can be readily translated to the care of acute HF patients in the ED. We identified few studies conducted in the ED setting; however, these examined the partial group of patients who were discharged without hospital admission.

Rame et al. [13] examined patients who were discharged home directly from the ED after presenting with ADHF, but then failed outpatient therapy within 90 days of discharge. In their analysis, the only predictor of outpatient treatment failure was increased respiratory rate at presentation. Miro et al. [7•] examined 259 patients and found that functional impairment was a predictor of recurrent presentation with ADHF within 30 days of discharge from the ED. They also found that a history of hypertension and systolic blood pressure greater than 160 mmHg on arrival predicted a decreased risk of return to the ED. In a retrospective analysis of 385 patients, Burkhardt et al. [15] found that blood urea nitrogen level over 30 mg/dL was the only factor that was associated with an increased risk of hospital admission.

In sum, there are few studies of HF patients who are discharged from the ED, and the majority of previously performed studies have been small. Prognostic factors from these small studies differ, and these individual prognostic factors are insufficiently sensitive or specific to guide clinical decision making. A unifying risk model for the broad range of acute HF patients presenting to the ED that incorporates the most important prognostic factors is potentially useful for guiding care.

## Extrapolating from Risk Stratification Models for Admitted Patients

While the prognostic factors predicting adverse outcomes among the broad spectrum of HF patients presenting to the ED have not been determined, partial insights may be obtained by examining the generally higher risk cohorts who have been admitted to hospital. There have been several risk stratification methods developed for hospitalized cohorts.

The Enhanced Feedback for Effective Cardiac Treatment (EFFECT) study investigators found that the predictors of 30-day mortality after admission for ADHF included older age, lower systolic blood pressure, higher respiratory rate, higher blood urea nitrogen level, and hyponatremia [16]. Subsequently published reports from the Outcomes of a Prospective Trial of Intravenous Milrinone for Exacerbations of Chronic Heart Failure (OPTIME-CHF) [17], the Acute Decompensated Heart Failure National Registry (ADHERE) [18], and others [19], have all confirmed the prognostic importance of systolic blood pressure, blood urea nitrogen and/or serum creatinine concentration, and hyponatremia for death occurring in hospital to 60 days after presentation. Additional predictors of mortality included New York Heart Association class IV symptoms [17], reduced ejection fraction, and low hemoglobin at admission [20], which were strong predictors of all-cause mortality from 30 to 60 days after HF presentation.

The challenge of identifying low-risk HF patients in the ED setting was exemplified by Chin and Goldman [21] who found that low initial blood pressure, high initial respiratory rate, hyponatremia, and new ST-T wave changes on the 12-lead electrocardiogram were associated with poor prognosis. However, the challenge of identifying low-risk HF patients in the ED setting was recognized because absence of the above factors did not identify a true low-risk group, since the rate of death or major complications was 6 % in the lowest risk group during the hospital stay [21]. The ADHERE classification and regression tree analysis identified a lower-risk group; however, in-hospital mortality still exceeded 2 % in the lowest-risk group, which may be unacceptably high for deciding who is safe to discharge home in the ED setting [18].

## Stratification of Risk among All Patients with ADHF in the ED

Few studies have been designed specifically to identify low-risk ED patients with ADHF. However, a risk stratification method that can identify low-risk patients may be of great value in the ED setting because low-risk patients could potentially be discharged home without hospital admission. In this section, we describe some of the published methods for acute HF risk stratification (Table 1).

**Table 1:** Emergency department–based prognostic factors for acute HF

	AHFI	SCP	AHCPR	EHMRG
Age				x
Female	x			
Systolic blood pressure	x	x	x	x
Oxygen saturation			x	x
Heart rate	x			x
Respiratory rate / tachypnea	x		x	
Temperature	x			
EMS transport				x
Troponin		x		x
Potassium	x			x
Creatinine/worsening renal function	x	x		x
Blood urea nitrogen	x	x		
Sodium	x	x		
White blood count	x			
Glucose	x			
Metolazone at home				x
Active Cancer				x
Prior myocardial infarction	x			
Angina	x		x	
Syncope			x	
Diabetes	x			
PCI procedure	x			
Chronic lung disease	x			
Significant peripheral edema			x	
Recent onset HF			x	
Concomitant acute medical illness			x	
Failure of outpatient management			x	
Altered mentation				
Significant arrhythmia				
ECG evidence of ischemia	x	x	x	
Arterial blood gas pH	x			
CXR: Pleural effusion	x			
CXR: Pulmonary congestion/edema	x		x	

*HF* heart failure; *AHFI* Acute Heart Failure Index; *SCP* Society of Chest Pain; *AHCPR* Agency for Health Care Policy and Research; *EHMRG* Emergency Heart failure Mortality Risk Grade; *EMS* Emergency medical services; *PCI* Percutaneous coronary intervention; *ECG* electrocardiogram; *CXR* Chest x-ray

Authors of the PROVIDE for HF study examined the association between components of the Agency for Health Care Policy and Research HF hospital admission criteria and rates of admission and mortality [9]. Presence of pulmonary edema, hypoxia not due to pulmonary disease, edema, and symptomatic hypotension or syncope were associated with increased probability of admission, longer duration of hospital stay, and 30-day mortality. While admitted patients did indeed have a higher mortality rate, it is important to note that this study found that clinical judgement was not always able to correctly distinguish patients at higher or lower risk of death, especially as the number of admission criteria increased [9]. The importance of acute respiratory status was affirmed by the examination of a nurse-rated triage acuity score that was objectively related to oxygenation status in a population-based study of 68,380 HF patients who visited the ED [22]. In this study, among those with triage categories that roughly corresponded to initial oxygen saturations below 90 %, 90 %–92 %, 93 %–95 %, and above 95 %, the associated 7-day mortality rates were 17.2 %, 5.9 %, 3.8 %, and 2.5 %, respectively [22].

### Society of Chest Pain Recommendations

Based on a review of the literature, the Society of Chest Pain published guidelines that are applicable to HF, which included a summary of high-risk features common to previously published risk stratification tools. The common high risk features were identified as low systolic blood pressure, renal impairment, hyponatremia, ischemic changes on electrocardiogram (ECG), and positive troponin. The society was careful to note that absence of high-risk features should not be interpreted as low risk without further investigation [23].

In a subsequent study, an external retrospective validation was performed using data from the multinational heart failure and Audicor technology (Inovise Medical, inc., Beaverton, OR) for rapid diagnosis and initial treatment (HEARD-IT) study [24•]. Using the guideline-based risk criteria, 20.2 % of the cohort was defined as non–high risk because they did not exhibit any high-risk features on presentation. The non–high risk group exhibited a 0.5 % mortality rate and 12.4 % cardiac event rate, and of the latter most were 30-day readmissions for HF. While those with high-risk features had a 14.8 % overall cardiac event rate (of which 5.7 % were deaths), there was no significant difference in event rates between the high-risk and non–high risk groups [24•].

### Acute Heart Failure Index

The acute heart failure index (AHFI) was developed to identify admitted HF patients at low risk of mortality or serious inpatient adverse events [25]. A tree-based model comprising 21 different factors incorporating demographic, historical, vital sign, laboratory, electrocardiographic, and radiographic components was developed. These variables include arterial pH, diabetes, respiratory rate, pulmonary congestion on chest imaging, pulse rate, creatinine concentration, ECG evidence of myocardial ischemia, ECG evidence of myocardial infarction, blood urea nitrogen, sodium concentration, history of percutaneous coronary intervention (PCI), white blood count, glucose concentration, history of angina, history of myocardial infarction, sex, potassium concentration, temperature, systolic blood pressure, history of chronic lung disease, and pleural effusion. The algorithm requires knowledge of the most extreme value of the predictor variable on the day before or the day of admission for risk determination. This tool identified a low-risk group comprising 17.2 % of the derivation cohort that had rates of 0.3 % for inpatient mortality and 1 % risk of serious medical complications during admission.

A validation phase involving a retrospective population of 8,384 patients subsequently identified 19.2 % of these patients as low risk with an inpatient mortality rate of 0.7 %, rate of serious medical complication during admission of 1.7 %, and a 30-day mortality rate of 2.9 % [26]. A prospective validation cohort study was recently published, demonstrating similar results, with a rate of primary outcome events of 1.7 % occurring in 23 % of the overall cohort who met low-risk criteria [27•]. While the AHFI has been well-validated in cohorts who have already been admitted to hospital, it has not been widely studied in a broad range of HF patients, including those who have been discharged from the hospital.

### Emergency Heart Failure Mortality Risk Grade

The Emergency Heart Failure Mortality Risk Grade (EHMRG) is unique compared to other validated risk prediction models because it was derived in a broad cohort of patients with ADHF presenting to the ED who were either discharged home or admitted to the hospital [28••]. The score was derived and validated in a cohort of 12,591 HF patients who were not palliative and presented to one of 86 EDs in a population-based study. The primary outcome was 7-day mortality, which is temporally close to the acute HF visit, and therefore clearly of importance to the physician in the ED. Prior studies have found that mortality among HF patients is primarily cardiovascular [29, 30], and therefore deaths will often be related to the cardiovascular disease with which the patients presented at the index ED visit.

**Fig. 1:**
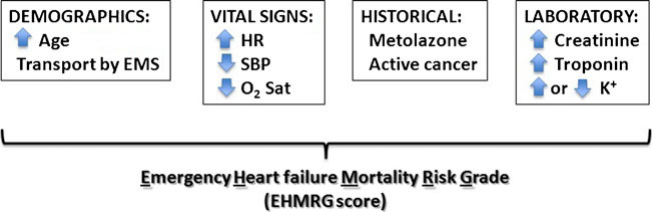
Variables comprising the EHMRG. *EMS* emergency medical services; *HR* heart rate; *SBP* systolic blood pressure

The score combines age, systolic blood pressure, heart rate, oxygen saturation, serum creatinine, serum potassium, serum troponin, presence of active cancer, current use of metolazone, and mode of arrival to the ED to estimate 7-day mortality after presenting to the ED with ADHF (Fig. 1). Dividing patients into quintiles of risk based on the EHMRG, the 7-day mortality rates were 0.3 % in the two lowest risk quintiles. The highest-risk quintile was further stratified into two groups corresponding to the highest two deciles of risk, with mortality rates of 3.5 % and 8.2 % at 7 days. Among those who were discharged, the odds ratios for death were greater than eightfold and 21-fold in the two highest risk groups compared to the two lowest risk quantiles combined. Similarly, the odds ratios for admitted patients were greater than ninefold and 23-fold in the highest two risk groups compared to the lowest two risk quantiles combined. There was an exceedingly low 7-day mortality rate of 0.2 % among those in the lowest two risk quantiles who were discharged from the ED, suggesting that risks may be further reduced if coupled with clinical judgement. Because the EHMRG incorporates information readily available to clinicians in the ED and is applicable to all patients with ADHF even before a disposition decision is made, the EHMRG score will likely be a helpful tool for the initial assessment of these patients (web calculator available at www.ccort.ca).

## Observation Units

Hospitals in various countries have integrated observation units into their EDs as an alternative to inpatient ward admission or direct discharge home. Common diagnoses in these units include chest pain, syncope, atrial fibrillation, asthma, and transient ischemic attacks. Patients moved to the observation unit typically remain under the care of staff from the ED with the goal of discharge home after a longer time of observed management. Implementation of such a unit in EDs varies by country. About one third of hospitals in the United States had dedicated observation units in 2007–2008 [31], but the proportion of hospitals with observation units in most other countries has not been reported.

Data from a tertiary care teaching hospital showed that implementation of an observation unit admission and treatment protocol for ADHF reduced rate of return to the ED with ADHF, mortality rate, and admissions to both the observation unit and inpatient unit for ADHF at 90 days [32]. It has since been suggested that a specialized HF observation unit may be best for patient care while reducing admission rates for ADHF [32]. However, others have reported that outcomes of 30-day readmission and recurrent ED visits for ADHF or mortality were similar when patients managed in an observation unit were compared to those who were hospitalized directly from the ED [33].

It has been found that observation units provide a cost-effective alternative compared to hospital admission for those with non–high risk HF [34••]. In the base case, compared to an ED discharge strategy, hospitalization had a very high marginal cost-effectiveness ratio of $684,101 per quality-adjusted life year, whereas observation unit admission exhibited a reasonable cost-effectiveness ratio of $44,249 per quality-adjusted life year [34••]. However, the cost-effectiveness of observation units is dependent on baseline risk, because with increasing risk of readmission or post-discharge adverse events, it becomes more cost-effective to admit to hospital, again emphasizing the importance of accurate risk stratification in the ED [34••]. Observation units also may represent an opportunity to better assess the functional capacity of patients, which may further assist in making clinical decisions, and determining the need for home supports for patients who are planned for discharge home [35].

A potential challenge is that not all HF patients are suitable candidates for management in an observation unit. Indeed, approximately 20 % of unselected patients who are managed in observation units are eventually admitted to the hospital [36, 37]. Low-risk patients who are being considered for discharge home are likely the best candidates for observation unit management. Diercks et al. [38] conducted a prospective study of a convenience sample of almost 500 patients with a diagnosis of ADHF in the ED. They defined low-risk patients by a length of stay less than 24 h and no adverse events of death, myocardial infarction, arrhythmia, or rehospitalization within 30 days. The low-risk group was found to have a systolic blood pressure greater than 160 mmHg and a normal serum troponin [38]. Prognostic risk algorithms for acute HF may better assist in the selection of low-risk patients for observation unit–based care because prior data suggest that many HF admissions may not be avoided by the nonselective use of observation units [36, 37].

## An Approach to Using EHMRG in Clinical Practice

**Fig. 2:**
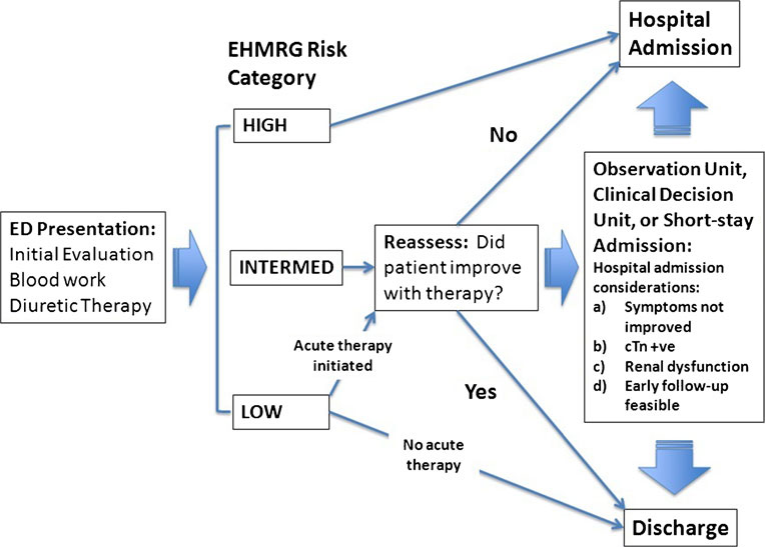
Using the EHMRG. *EHMRG* Emergency Heart Failure Mortality Risk Grade; *ED* emergency department

The precise role of the observation unit for different categorizations of the EHMRG has not been fully defined. The EHMRG risk algorithm [28••] could be adapted for use in conjunction with observation units, clinical decision units, or short-stay hospital beds, as proposed in Fig. 2. In the absence of extenuating circumstances, those with a high-risk EHMRG score should be admitted to hospital. Intermediate-risk patients who do not improve symptomatically should be admitted, and conversely, those who improve could be considered for discharge. Admission to the observation unit or a short-stay hospitalization may be potential options for intermediate-risk patients in whom the discharge disposition is not clear. Low-risk patients who were administered therapy in the ED should be reassessed for symptomatic improvement. However, low-risk patients who did not require any therapy could be considered for direct discharge from the emergency discharge.

## Conclusions

As the burden of HF increases worldwide, the ED will become an increasingly important focal point. As the gatekeeper of hospital-based resources, decisions made in the ED setting are also of critical importance. Indiscriminate hospitalization of the majority of HF patients, including those who are low risk, will have major fiscal implications that may not be sustainable. Hospital resources should be utilized to a greater extent for higher-risk patients, while lower-risk patients who are dischargeable could ideally be managed during the transitional phase via rapid outpatient care programs. The availability of validated risk stratification tools will be instrumental in aiding clinicians’ decision-making in this regard.

Risk stratification tools will likely operate in a complementary way with other management strategies, such as observation units and rapid follow-up cardiac clinics that can assess HF patients who have been recently discharged from the ED. Finally, it is conceivable that future studies may be able to deconstruct the key benefits of hospital-based care. For example, hospitalized patients experience more rapid assessment of left ventricular function and more careful titration and management of medications. If these important processes of care can be provided rapidly and routinely to patients after ED discharge, the threshold of risk for deciding which HF patient may benefit from hospital admission or discharge home may be altered. The use of risk stratification methods and novel models of early ambulatory care delivery may substantially improve the efficiency of medical care of patients with acute HF syndromes while optimizing utilization of health care resources.
